# Value Proposition to a Hospital of Obtaining 36-inch Standing Scoliosis Film Technology

**DOI:** 10.7759/cureus.3044

**Published:** 2018-07-24

**Authors:** Russell D Parks, Richard P Menger, Andrew Zhang, Anthony Sin

**Affiliations:** 1 Louisiana State University Health Sciences Center School of Medicine, Shreveport, USA; 2 Department of Neurosurgery, Louisiana State University Health Sciences Center, Shreveport, USA; 3 Department of Orthopaedic Surgery, Louisiana State University Health Sciences Center, Shreveport, USA; 4 Department of Neurosurgery, Louisiana State University Health Sciences Center School of Medicine, Shreveport, USA

**Keywords:** 36 inch standing films, spinal deformity, radiographs, value proposition

## Abstract

Introduction

Some hospitals do not have the technological capabilities of obtaining full 36-inch long-standing films to evaluate patients via proper sagittal balance spinal imaging protocol. Resistance from hospital administration for the purchase of proper hardware and software remains frustrating for spinal surgeons at both community and academic hospitals.

Materials and methods

Recurring transaction-based revenue streams were applied comparing cost with the different income generation at the hospital level. Cost is fixed cost, attributed to purchasing both the physical radiograph machine as well as the necessary software capabilities. Marginal cost was negligible as both materials and human capital are largely fungible and trivial at the margin. Revenue generation is largely identical to marginal revenue. Income was linked to the Hospital Outpatient Prospective Payment System for radiographic interpretation of films (Current Procedural Terminology (CPT) 72069). Income was also estimated from surgical volume calculation.

Results

The listed prospective outpatient radiographic reimbursement for the hospital was $24.36 per film. Medicare-defined reimbursements for a complex spinal fusion except cervical with spinal curvature, malignancy or 9+ fusions with a Major Complication or Comorbidity (MCC) was listed at $55,228, and with a Complication or Comorbidity (CC) was noted to be $40,566. Complex spinal fusion except cervical with spinal curvature, malignancy or 9+ fusions without CC/MCC was listed as $30,913. Lumbar spinal fusion except cervical with MCC was $39,164 and with CC was $23,490. University Neurosurgery at Louisiana State University (LSU) Health Sciences Center in Shreveport, LA performed 1,013 thoracolumbar procedures in fiscal year (FY) 2015 with 557 (54.9%) being instrumented procedures. At a minimum, all instrumented procedures could benefit from proper spinal axis imaging, representing $13,568.52 of transaction-based annual gross revenue from radiographs alone. Hypothetical revenue generation of $491,696.42 was calculated.

Conclusion

There is a significant value proposition to the hospital in obtaining the proper technology for formal standing 36-inch scoliosis imaging. Marginal cost is negligible, while there are significant opportunities for marginal revenue per image obtained through transaction-based gross revenue, as well an immense hypothetical revenue stream from surgery-related gains. More importantly, it ensures a proper and complete delivery of spinal health to the hospital's healthcare population.

## Introduction

Spinal deformity affects 2–3% of the U.S. population, accounting for 6–9 million individuals [1] and $86 billion in annual costs [2]. Many of these individuals will require bracing or surgical correction for their deformities. Of those who receive surgical correction, 27% will be readmitted due to complications or need for further correction, making spinal deformity a major economic burden. The average total hospital cost for surgical correction of spinal deformities in adults exceeds $120,000, with the primary surgery accounting for over $103,000, on average. An additional $67,262 is the average cost for those individuals requiring readmission, increasing the total cost of their spinal correction by more than 70% [3]. Minor deformities that do not require surgical correction can be treated with bracing and other non-surgical means, but this does not come without significant expenses as well. Some braces can cost upwards of $5,000 [1] and some studies have found that certain patient populations undergoing non-surgical treatment for their spinal deformities can pay over $14,000 in the first two years alone [2]. Thus, the high morbidity and costs associated with spinal deformity make accurate diagnoses and careful patient selection highly important.

Paramount to proper management of these deformities is appropriate diagnosis. Full-length 36-inch standing radiographs are helpful for a full evaluation of the entire spine in patients with scoliosis. Even when the abnormality is limited to the cervical or thoracic spine, lumbar and pelvic images are often also required for a full understanding of the patient’s deformity. Thirty-six-inch standing films eliminate the need for further dedicated imaging of the spine, including patients with cervical scoliosis. Most importantly, 36-inch complete spine films enable accurate calculation of sagittal balance [4]. However, some hospitals do not have the technological capabilities of obtaining full 36-inch long-standing films to evaluate patients via proper sagittal balance spinal imaging protocol. Resistance from hospital administration for the purchase of proper hardware and software remains frustrating for spinal surgeons at both community and academic hospitals. The purpose of this study is to present an objective analysis and data surrounding the economics of a standing scoliosis film to a single institution.

## Materials and methods

Recurring transaction-based revenue streams were applied comparing cost with the different income generation at the hospital level. Cost is fixed cost, attributed to purchasing both the physical radiograph machine as well as the necessary software capabilities. Marginal cost was negligible as both materials and human capital are largely fungible and trivial at the margin. Revenue generation was largely identical to marginal revenue. Income was linked to the Hospital Outpatient Prospective Payment System for radiographic interpretation of films (Current Procedural Terminology (CPT) 72069) [5]. Income was also estimated from surgical volume calculation.

No Internal Review Board was necessary.

## Results

The listed prospective outpatient radiographic reimbursement for the hospital was $24.36 per film. Medicare-defined reimbursement for a complex spinal fusion except cervical with spinal curvature, malignancy or 9+ fusions with a Major Complication or Comorbidity (MCC) was listed at $55,228, and with a Complication or Comorbidity (CC) was noted to be $40,566. Complex spinal fusion except cervical with spinal curvature, malignancy or 9+ fusions without CC/MCC was listed as $30,913. Lumbar spinal fusion except cervical with MCC was $39,164 and with CC was $23,490 [6].

University Neurosurgery at Louisiana State University (LSU) Health Sciences Center in Shreveport, LA performed 1,013 thoracolumbar procedures in Fiscal Year (FY) 2015 with 557 (54.9%) being instrumented procedures [7]. At a minimum, all instrumented procedures could benefit from proper spinal axis imaging, representing $13,568.52 of transaction-based annual gross revenue from radiographs alone. Hypothetical revenue generation of $491,696.42 is found in Table 1.

**Table 1:** Hospital-based recurring transaction-based venue stream for 36-inch standing scoliosis radiographs. *AR = TR/q, assumption is that marginal revenue MR = AR, (MR = ΔTR/Δq) ** TR = Price*q, substituted as AR *q ***Per Mehta et al. [8] in Neurosurgery, nearly 50% of degenerative spines remain unbalanced. ****Conservative estimate puts 1/10 patients would have a long segment surgery; a hospital performing 1000 spine surgeries per year would perform a long segment fusion every other week. & Difference between spinal fusion except cervical with spinal curvature, malignancy or 9+ fusions with co-morbidity (CC) spinal fusion except cervical with CC is $17,076.

Hospital-based Recurring Transaction-based Venue Stream for 36-inch Standing Scoliosis Radiographs				
Clinical entity	Average Revenue* (AR)	Unit amount (q)	Increase in Total Revenue per clinical entity** (TR)	Gross Revenue
36-inch standing radiograph	$24.36	557	$13,568.52	
# of instrumented spine cases in	/	557	/	
# of patients in sagittal imbalance***	/	278	/	
Increased surgical output****	/	28	/	
Increase reimbursement w/ long segment fusion&	$17,076	28	$478,128	
				$491,696.52

## Discussion

Routine imaging obtained for the complete evaluation of the spine in cases of scoliosis often entails “full-spine radiography”, “full” or “long-cassette” radiographs, or “total spine X-rays” [9-11]. These terms refer to the use of a 36-inch cassette to capture posteroanterior (PA) and lateral X-rays that typically span the occiput to the bilateral femoral heads [9]. The patient is asked to stand approximately 72 inches away from the film to decrease the distortion and to ensure proper magnification. Differing densities within the thoracic and lumbosacral region will often require graduated filtering of the beam to allow for adequate image penetration. By convention, PA radiographs are viewed with the heart on the left side and lateral radiographs will have the patient facing towards the right.

Aside from proper equipment, patient positioning is also of utmost importance with 36-inch standing X-rays. As its name implies, the patient is asked to weight bear for these images as the deformities are more pronounced in this position. The patient’s legs, including the hips and knees, should be fully extended with the feet at shoulder width. To minimize potential extraneous sources of compensation of spinal deformity, patients are asked to maintain a free-standing position when obtaining these radiographs. Horton et al. examined various positioning techniques for these 36-inch cassette radiographs and concluded that patients should rest their hands in the clavicle position, with their elbows fully flexed, hand in a relaxed fist posture with the wrists flexed and the proximal interphalangeal joints lying within the supraclavicular fossae [12]. Any clinically significant leg length discrepancy should be addressed by using blocks to ensure a level pelvis, which should then be annotated on the X-ray.

**Figure 1:**
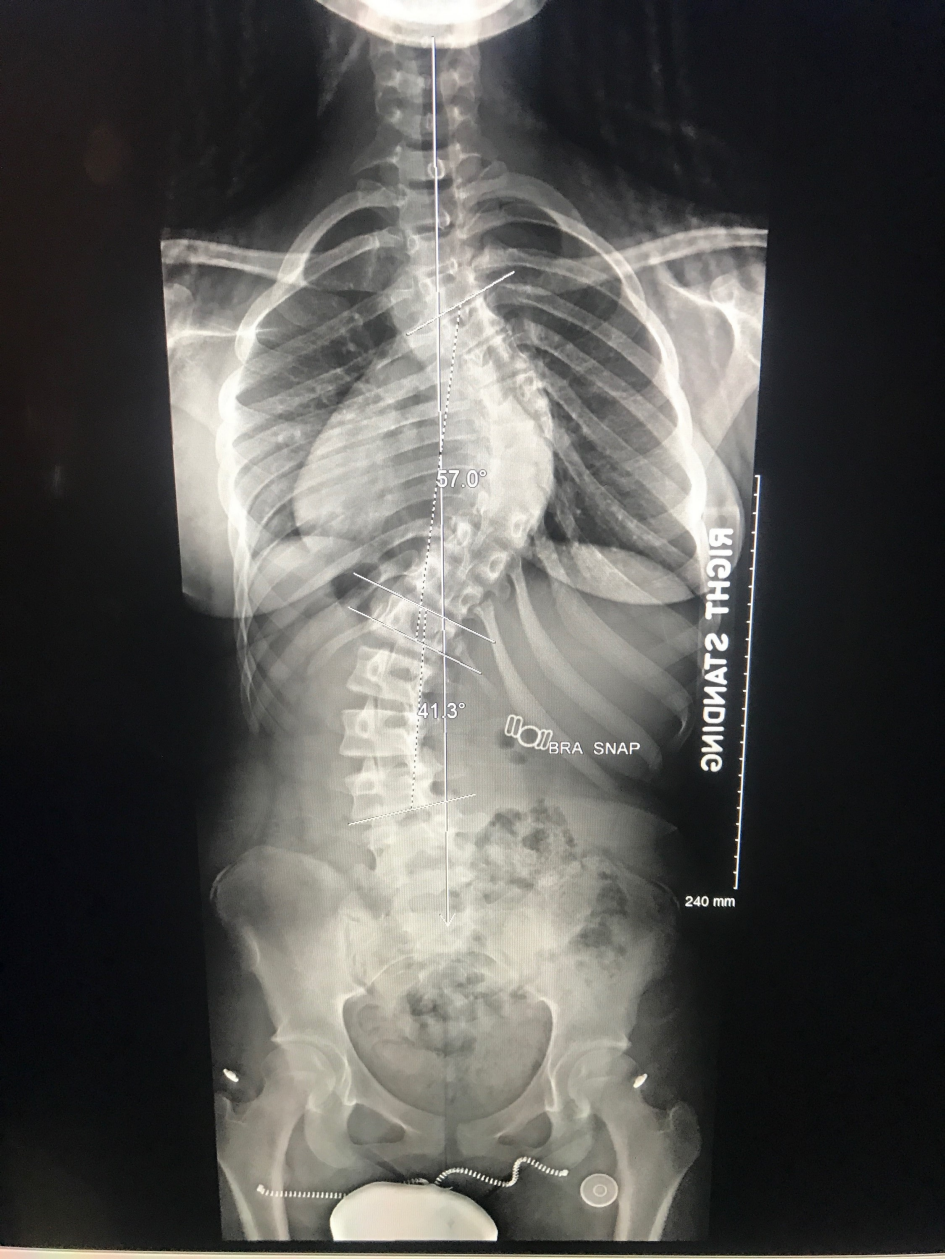
Preoperative posteroanterior (PA) 36-inch standing scoliosis radiograph.

**Figure 2:**
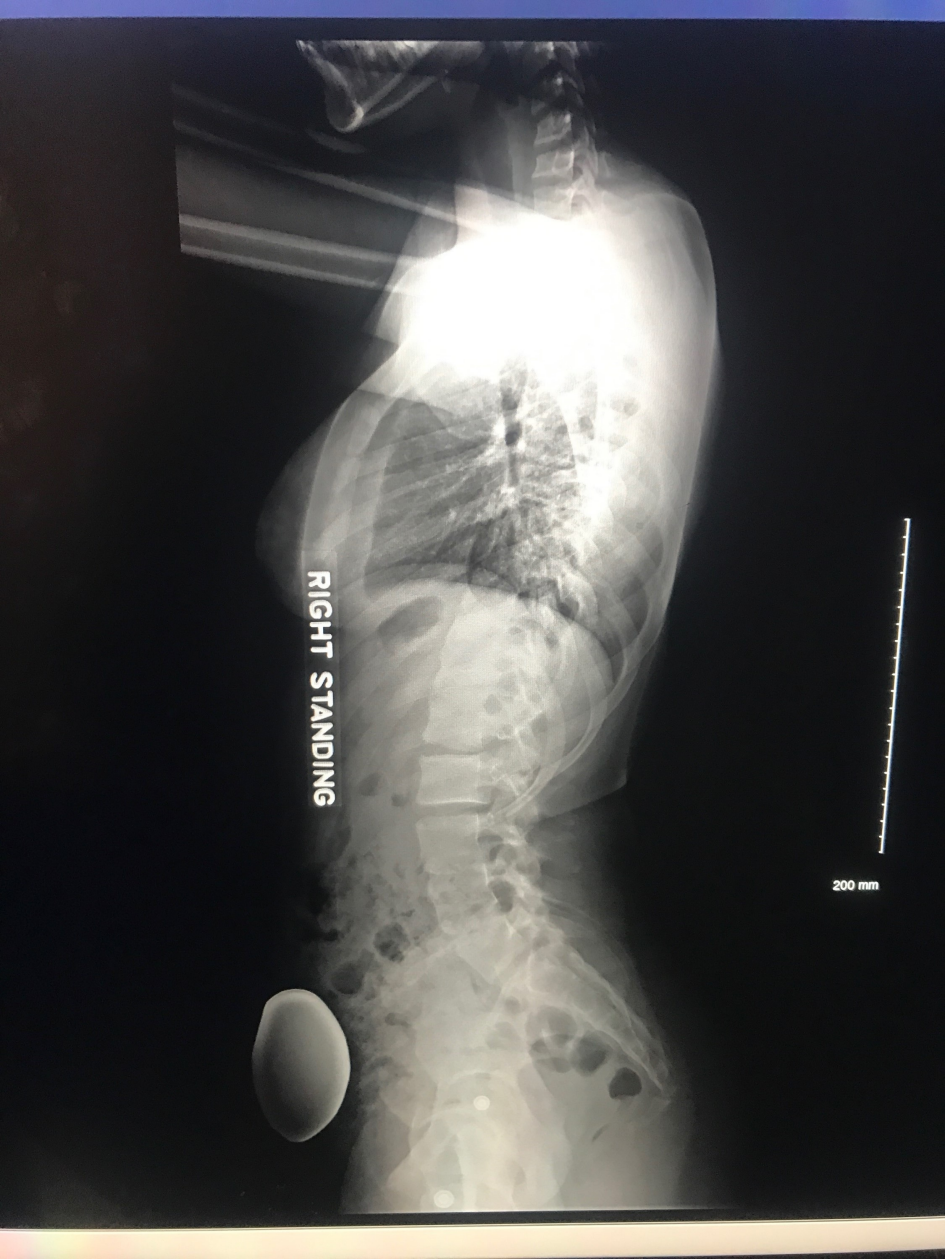
Preoperative lateral view of 36-inch standing scoliosis radiograph.

**Figure 3:**
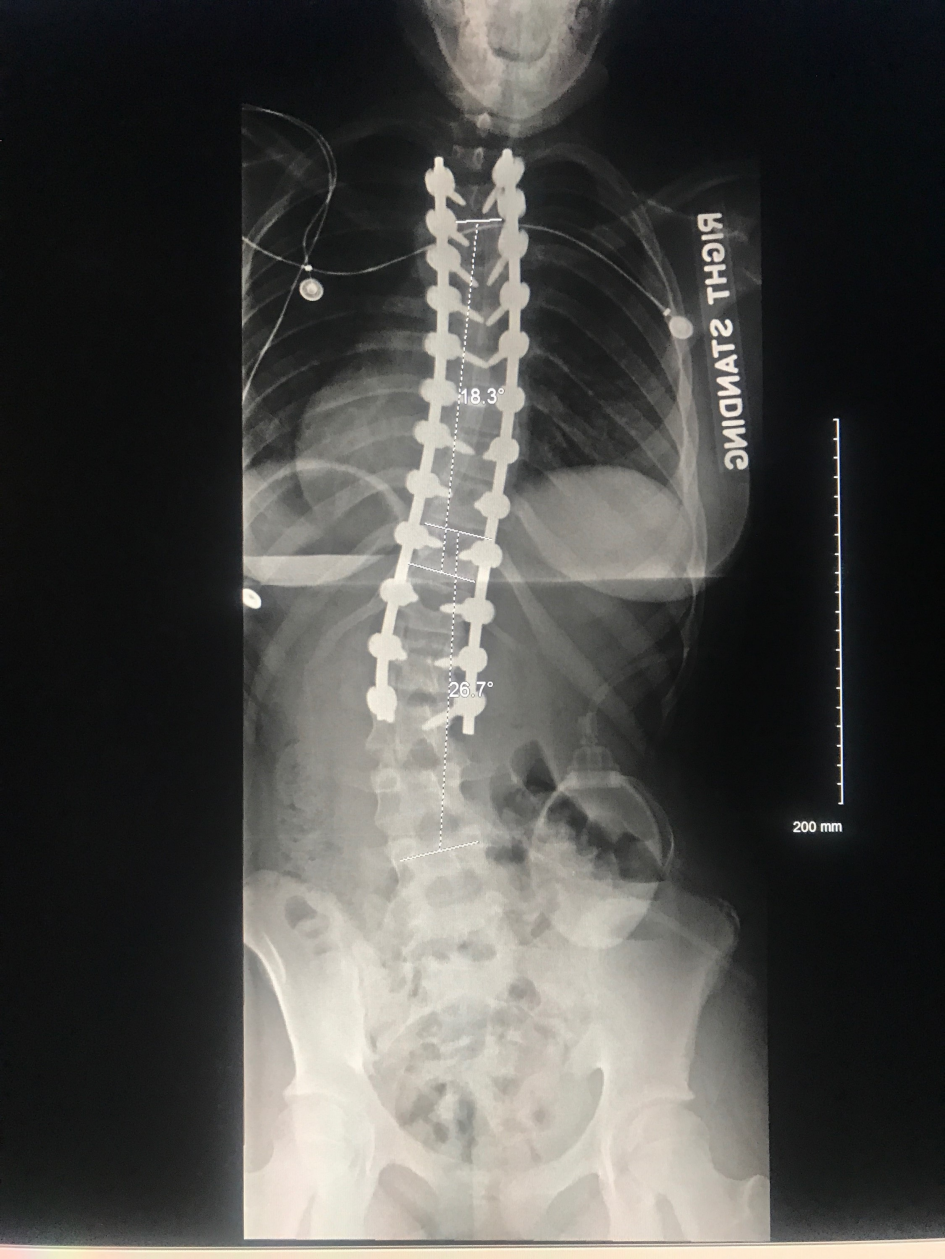
Postoperative posteroanterior (PA) 36-inch standing scoliosis radiograph.

**Figure 4:**
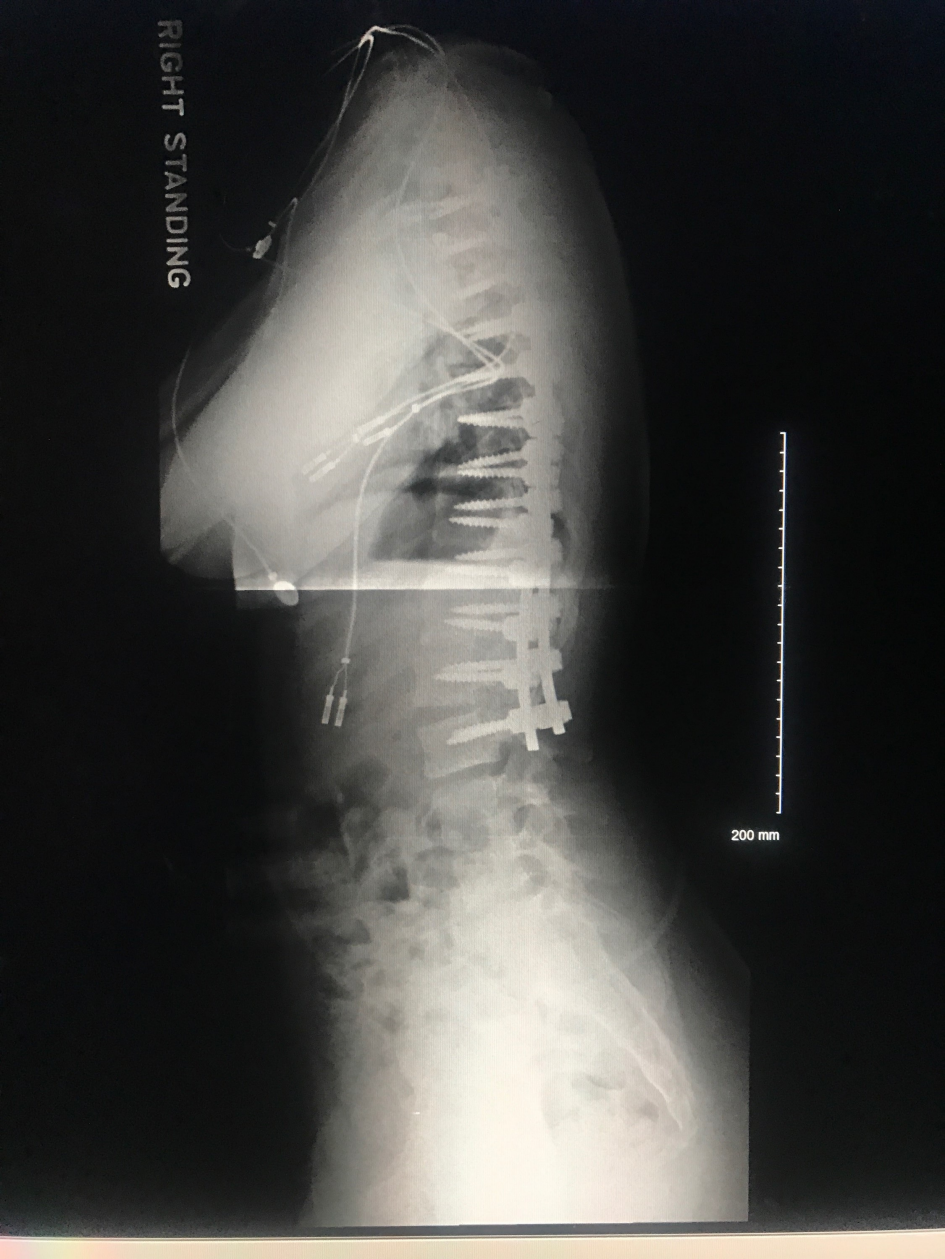
Postoperative lateral view of 36-inch standing scoliosis radiograph.

Thirty-six inch standing films are essential for proper scoliosis evaluation. Piecemeal images of the thoracic or lumbar spine will often fail to comprehensively depict the sagittal and coronal deformity of the spine. Full-spine radiography is also necessary for accurate assessment of the cervical spine as well as spinopelvic alignment [10]. Examples of pre-operative and post-operative X-rays can be seen in Figures 1-4.

Radiographic features of sagittal balance

One of the key radiographic measurements in predicting health status and long-term outcomes of adult spinal deformity is sagittal balance of the spine. Sagittal balance is determined by the location of the vertical plumb line (intersecting the center of the C7 vertebral body) in relation to the posterosuperior aspect of the S1 endplate. A positive sagittal imbalance will have a plumb line more than 5 to 6 centimeters (cm) anterior to the posterosuperior aspect of the S1 endplate, while a plumb line posterior to this landmark denotes a negative sagittal imbalance [13]. Positive sagittal imbalance has been found to be the most significant predictor of adverse health outcomes in patients with spinal deformity. Notable outcomes associated with positive sagittal imbalance include decreased activity level, increased pain, and a higher degree of disability. Establishing the presence of sagittal imbalance preoperatively, and confirming its correction postoperatively, has been shown necessary in improving these clinical outcomes. Established protocol for measuring sagittal balance requires coronal and sagittal 36-inch standing radiographs [14]. Therefore, this specific radiograph technology is required for optimal care to be provided to patients with spinal deformity.

Importance of pelvic parameters

Sagittal balance relies on the distribution of weight load between the spine, pelvis, and lower extremities. Due to the articulation of the lumbar spine with the pelvis at the sacral plateau, the orientation of the pelvis and its ability to rotate around the axis of the femoral heads will determine the underlying sagittal profile [15]. Pelvic orientation is described by the sacral slope, pelvic tilt, and pelvic incidence. Sacral slope (SS) refers to the angle between a horizontal line and the plane of the sacral plateau [13, 15], with 40° considered normal (range 20°–65°) [13]. Pelvic tilt (PT) refers to the angle between a vertical line originating at the center of the head of the femur and a straight line from the head of the femur to the midpoint of the S1 endplate [13, 15], with 12° considered normal, with concern for pelvic tilt greater than 20° [13]. Retroversion of the pelvis around the posterior aspect of the femoral heads leads to an increased PT. As PT increases, the orientation of the sacral plateau becomes more horizontal and extension at the hip is limited. Likewise, anteversion of the pelvis around the anterior aspect of the femoral heads leads to a decreased PT, increasingly vertical sacral plateau, and limitation of hip flexion [15].

Pelvic incidence (PI) refers to the angle between a line perpendicular to the plane of the sacral plateau originating at the midpoint of the upper S1 endplate and a straight line from the head of the femur to the midpoint of the upper S1 endplate [13, 15], with 52° considered normal (range 35°–85°). PI can simply be defined as the sum of the SS and PT (PI = SS + PT). Due to the fusion of the pelvic bones at the end of growth, PI is a fixed value, but can be variable from patient to patient. Since PI is stable for a given individual, the PT and SS are inversely proportional to each other. PT is ideally <50% of the PI, making SS ideally >50% of the PI. Higher values of PI and SS lead to dramatic increases in the natural inward curvature of the lumbar spine, also known as lumbar lordosis (LL) [15]. Therefore, due to the extreme variations in pelvic orientation from patient to patient, obtaining proper imaging capabilities, such as 36-inch film technology, to measure the sagittal balance becomes necessary in the preoperative evaluation of spinal deformity.

Imaging and patient selection

Patient selection is one of the most relevant factors to successful scoliosis surgical outcomes. It is an expensive surgery and having proper imaging can ensure appropriate selection of operative candidates. When considering a patient for spinal surgery, cost-effectiveness must be taken into account. Acceptable cost-effectiveness has been defined as less than $100,000 per quality-adjusted life year (QALY). Terran et al. [2] found a total of 40.7% of patients who underwent spinal deformity surgery to qualify as cost-effective at five years postoperatively. Evaluation of the same population at two years postoperatively found only 9% to be cost-effective. This can be contributed to the high upfront direct care costs associated with spinal surgery. However, since spinal surgery is known to be a durable long-term treatment, its cost-effectiveness rises with each postoperative year. Common characteristics of patients determined to be qualified as cost-effective at five years included older age, worse Scoliosis Research Score (SRS) in pain and activity, worse baseline Oswestry Disability Index (ODI) score, and deformities requiring correction of less than eight vertebral levels [2]. Proper imaging, along with evaluation for these predicting characteristics, leads to a better understanding of which patients will likely qualify as cost-effective and have favorable outcomes after spinal surgery.

Without means to 36-inch standing films, institutions may not be providing optimal care to patients with spinal deformity. Not being able to identify and manage sagittal imbalance can negatively afflict patients. In fact, patients with sagittal imbalance were found to have worse Short Form 36 (SF-36) Physical Component scores than individuals suffering from visual impairment or limited use of their upper and lower extremities [16]. Untreated or postoperative development of sagittal imbalance leads to increased pain, disability, negative self-image, and decreased social functioning [8, 13], as well as a higher rate of pseudoarthrosis, implant failure, and adjacent vertebral disease. All of these components make restoration of sagittal balance crucial for any spinal surgery. Certain predisposing factors, such as high PI, low SRS scores, and lower degree of lumbar lordosis, have been shown to increase the risk of developing sagittal imbalance after surgery [8]. It is necessary to monitor for development of sagittal imbalance after spinal surgery. McDowell et al. [17] found that sagittal imbalance worsens within a week of surgery, but then gradually improves from six weeks to one year. They determined that serial imaging is needed past six months to assess for successful restoration of sagittal balance. Without such imaging technology, sagittal balance cannot be assessed to determine whether it has been restored and maintained after surgery.

## Conclusions

There is a significant value proposition to the hospital in obtaining the proper technology for formal standing 36-inch scoliosis imaging. Marginal cost is negligible, while there are significant opportunities for marginal revenue per image obtained through transaction-based gross revenue, as well an immense hypothetical revenue stream from surgery-related gains. More importantly, it ensures a proper and complete delivery of spinal health to the hospital’s healthcare population.
